# Changes in health-related quality of life (EQ-5D) dimensions associated with community-based musculoskeletal physiotherapy: a multi-centre analysis

**DOI:** 10.1007/s11136-018-1883-7

**Published:** 2018-06-09

**Authors:** N. Caplan, H. Robson, A. Robson, M. Kelly, G. Wilkes

**Affiliations:** 1 Faculty of Health and Life Sciences, Northumbria University, Newcastle upon Tyne, NE1 8ST UK; 2 Connect Health, Newcastle upon Tyne, UK

**Keywords:** Rehabilitation, Intervention, Physical therapy, Quality of life, Musculoskeletal

## Abstract

**Purpose:**

To determine the changes in each of the five dimensions of the EuroQol 5-dimension index associated with community-based physiotherapy.

**Methods:**

Four thousand one hundred and thirty-six patients that received community-based musculoskeletal physiotherapy across five NHS centres completed the EQ-5D on entry into the service and upon discharge. Patients were categorised on symptom location and response to treatment based on their EQ-5D index improving by at least 0.1 (“EQ-5D responders”). For each symptom location, and for responders and non-responders to treatment, the mean (± SD) were calculated for each dimension pre- and post-treatment as well as the size of effect.

**Results:**

The mobility dimension improved (*p* < 0.05) in all symptom locations for EQ-5D responders (*d* = 0.26–1.58) and in ankle, knee, hip and lumbar symptoms for EQ-5D non-responders (*d* = 0.17–0.45). The self-care dimension improved (*p* < 0.05) in all symptom locations for EQ-5D responders (*d* = 0.49–1.16). The usual activities dimension improved (*p* < 0.05) across all symptom locations for EQ-5D responders (*d* = 1.00–1.75) and EQ-5D non-responders (*d* = 0.14–0.60). Despite the pain/discomfort dimension improving (*p* < 0.05) across all symptom locations for both EQ-5D responders (*d* = 1.07–1.43) and EQ-5D non-responders (*d* = 0.29–0.66), the anxiety/depression dimension improved (*p* < 0.05) from higher starting levels in EQ-5D responders (*d* = 0.76–1.05) with no change seen for EQ-5D non-responders (*d* = − 0.16 to 0.06).

**Conclusions:**

Clinicians should not assume that a patient presenting with pain but expressing high anxiety/depression is unlikely to respond to treatment, as they may show the best HRQoL outcomes. For patients presenting with pain/discomfort and low levels of anxiety/depression, the EQ-5D index is perhaps not a suitable tool for sole use in patient management and service evaluation.

## Introduction

Health-related quality of life (HRQoL) outcomes are often used to evaluate healthcare interventions [1] and service performance [2] and have been suggested as being useful for clinicians in guiding treatment planning [2, 3]. Whilst the traditional goal of musculoskeletal physiotherapy is to improve function and reduce pain, these would be expected to have a key influence on quality of life. The EuroQol 5 Dimensions (EQ-5D) assess HRQoL across five dimensions, including mobility, self-care, usual activities, pain/discomfort and anxiety/depression [4]. Each dimension is scored from one to five, with one being the best possible score. The scores from all five dimensions are then combined and scaled, based on national norms, to provide an index that represents overall HRQoL. In the UK, this index ranges from − 0.594 to 1, with 1 the best possible quality of life and values below zero indicating quality of life worse than death [5].

In musculoskeletal healthcare, EQ-5D has been used to evaluate services such as aquatic exercise therapy [6], postural exercise [7], telephone-based physiotherapy [8], back and neck pain interventions [9], acupuncture [10, 11] and advanced physiotherapy services [12–14], with improvements ranging from 0.048 [12] up to 0.20 [9]. In community-based physiotherapy, (i.e. received by patients from the general population living independently), Harding et al [15] reported improvements in EQ-5D index of 0.25 in patients allocated to an intervention to reduce waiting times compared to improvements of 0.18 in patients following standard care pathways. More recently, we reported improvements in EQ-5D index associated with community-based physiotherapy of 0.203 [16].

Whilst an overall measure of HRQoL using the EQ-5D index is useful for evaluating the effectiveness of community-based physiotherapy intervention, no studies have explored changes in the individual dimensions of EQ-5D that contribute to the EQ-5D index. By investigating how each dimension interacts over the course of physiotherapy management, and how these changes differ based on the anatomical site of pain/injury, useful information could be derived to predict outcome of physiotherapy intervention. This study aimed to determine the association between community-based physiotherapy and changes in EQ-5D dimension scores in terms of their relative contribution to improved HRQoL.

## Methods

### Population

Patients receiving community-based physiotherapy from one of the five National Health Service centres (Camden, Gateshead, Newcastle, Northumberland, South West Essex) between January 2012 and April 2016 were eligible to be included in the sample. All patients were registered with a general practitioner (GP). Entry to the service was via referral from their GP, or through self-referral via a telephone assessment and advice service. For those patients that could not use the self-referral service (i.e. patients aged less than 16 years, or those with hearing impairments) could only enter the service through GP referral. Patients entering the service completed a pre-intervention EQ-5D. Those unable to understand the English language sufficiently to answer the questions without the need for an interpreter did not complete a pre- or post-intervention EQ-5D and were excluded from this study, due to only the English language version of the EQ-5D being licenced for use within the service. The mean new-to-review ratio across these centres within this period was 1:2.38. The patient population used in this study was the same as reported on previously [16].

### Intervention

On first entry into the service, patients were categorised according to symptom location. Thirteen symptom location categories were used, including the foot, ankle, knee, hip, sacroiliac (region where the spine meets the pelvis), lumbar (lower back), thoracic (middle-upper back), neck, temporomandibular (jaw), shoulder, elbow, wrist, hand, as well as generalised pain and “other disorders not otherwise stated”. Physiotherapy-based management was subsequently provided to patients in a community setting, appropriate to their symptoms. Patients received interventions that typically included lifestyle advice, exercise therapy, manual therapy, taping, soft tissue techniques, electrotherapy and/or acupuncture. The most appropriate intervention was determined according to patient need, evidence-based practice and national and local clinical guidelines, using shared decision making. Episodes of care usually involved an initial telephone triage assessment and treatment assessment followed by face-to-face appointments, if required.

### Ethical approval statement

The study was approved by the Northumbria University Ethics Committee as a retrospective study.

### Outcomes

The EQ-5D was administered upon first entry into the physiotherapy service and was subsequently requested from patients at discharge following the completion of treatment. Within each of the five dimensions, a patient will receive an integer score from 1 to 5, where a score of 1 indicates the best outcome (full health) for that dimension (Table 1). From the individual dimension scores, an index is calculated that takes into account national population norms (national tariff). As this study evaluated EQ-5D data from a UK population, the UK tariff was used to calculate the EQ-5D index [5], which can range from − 0.594 to 1.0. Patients were categorised as either being “EQ-5D responders” to treatment in terms of HRQoL or “EQ-5D non-responders”, based on whether they showed an improvement in overall EQ-5D index of greater than 0.1, or not, in line with previous studies [16, 17].

**Table 1:** The five levels within each of the five dimensions of EQ-5D

Dimension/question	Score
Mobility	
I have no problems in walking about	1
I have slight problems in walking about	2
I have moderate problems in walking about	3
I have severe problems in walking about	4
I am unable to walk about	5
Self-care	
I have no problems washing or dressing myself	1
I have slight problems washing or dressing myself	2
I have moderate problems washing or dressing myself	3
I have severe problems washing or dressing myself	4
I am unable to wash or dress myself	5
Usual activities	
I have no problems doing my usual activities	1
I have slight problems doing my usual activities	2
I have moderate problems doing my usual activities	3
I have severe problems doing my usual activities	4
I am unable to do my usual activities	5
Pain/discomfort	
I have no problems pain or discomfort	1
I have slight problems pain or discomfort	2
I have moderate problems pain or discomfort	3
I have severe problems pain or discomfort	4
I have extreme pain or discomfort	5
Anxiety/depression	
I am not anxious or depressed	1
I am slightly anxious or depressed	2
I am moderately anxious or depressed	3
I am severely anxious or depressed	4
I am extremely anxious or depressed	5

### Data handling

The investigators had full access to the data from all five centres, which were recorded in a single nationwide system. Data were only included for patients that were being treated for a single morbidity, where the length of time in the service was between 2 and 16 weeks. This was to ensure that only data for a single treatment intervention were included, being deemed the minimum and maximum duration that a patient would remain in the service for a single bout of treatment. Given a study period of 4 years, some patients re-entered the service at a later date for a further assessment and treatment, and these were included in the study as separate records. Any duplicate records were removed prior to analysis. Patients were only included if their records showed both pre- and post-treatment EQ-5D index and individual dimension scores. Patients were excluded if they were referred to advanced services (CATS), as those patients had their post-treatment EQ-5D within CATS which, therefore, did not make this a valid representation of community-based physiotherapy management. Sacroiliac pain, temporomandibular pain and general pain categories were removed from the dataset due to having very low patient numbers, and the other disorders not otherwise stated category was also removed as it did not pertain to any specific symptom location.

### Data analysis

For each symptom location, mean (± SD) score for each of the five EQ-5D dimensions were calculated before and after treatment. Paired samples t tests were used to determine the significance of any difference between pre- and post-treatment. Cohen’s d effect sizes were also calculated for each pairwise comparison to provide an indication of the magnitude of effect between pre- and post-treatment. Cohen’s d effect sizes were calculated by the change in mean between pre- and post-treatment divided by the mean standard deviation. Effects were defined as trivial (*d* < 0.20), small (0.20 < *d* < 0.5), moderate (0.5 < *d* < 0.8) or large (*d* > 0.8).

## Results

Overall, 33,117 patient records were obtained. After removal of duplicates (*n* = 4832), 28,285 patients had a first EQ-5D recorded, of whom 5547 patients also had an end of episode EQ-5D recorded, who were included in the analysis. Those without a follow-up score (*n* = 22,275) were either discharged after their telephone assessment only as a one-stop shop, or failed to return the EQ-5D score after discharge if seen face to face. Four hundred and sixty-five patients were excluded as they returned more than two EQ-5D scores, as the data management system only retaining the first and last score. Of the 5547 patients with both a pre- and post-treatment EQ-5D, a further 1275 were excluded as they did not meet the remaining inclusion criteria. Due to very small patient numbers, those with temporomandibular pain (*n* = 4), generalised pain (*n* = 4), sacroiliac pain (*n* = 24) and other disorders not otherwise stated were excluded. This left a final sample for analysis of 4112 patient records.

The mobility dimension showed significant improvement across all symptom locations in EQ-5D responders. Large improvements were observed for lower limb joints and lumbar spine in EQ-5D responders. Moderate improvement in the mobility dimension was seen for all other symptom locations in EQ-5D responders except the elbow and hand which both showed small effect magnitudes (Table 2). EQ-5D non-responders to treatment still showed significant improvements in the mobility dimension for ankle, knee, hip and lumbar symptoms, although these changes were only trivial to small in magnitude. Pre-treatment mobility dimension scores ranged between 1.31 and 2.51 for EQ-5D responders and between 1.07 and 2.00 for EQ-5D non-responders.

**Table 2:** Pre- and post-treatment scores for the mobility dimension of the EQ-5D for patients that reported overall improvements in EQ-5D index and those that did not

	*n*	Pre-treatment		Post-treatment		Mean difference	*d*	95% confidence interval		*t*	df	*p*
		Mean	SD	Mean	SD			Lower	Upper			
Responders (EQ-5D index change from baseline > 0.1)												
Lower limb												
Foot pain	75	2.51	1.04	1.25	0.55	− 1.26	1.58	1.03	1.47	11.307	74	< 0.001
Ankle pain	141	2.13	0.92	1.18	0.44	− 0.95	1.40	0.81	1.10	13.082	140	< 0.001
Knee pain	538	2.12	0.91	1.17	0.44	− 0.95	1.41	0.88	1.02	26.411	537	< 0.001
Hip pain	188	2.10	0.94	1.21	0.54	− 0.89	1.20	0.77	1.01	14.571	187	< 0.001
Trunk and head												
Lumbar pain	690	2.06	0.99	1.20	0.51	− 0.86	1.15	0.80	0.93	25.588	689	< 0.001
Thoracic pain	94	1.32	0.66	1.07	0.26	− 0.25	0.54	0.14	0.35	4.542	93	< 0.001
Neck pain	382	1.48	0.81	1.09	0.35	− 0.39	0.67	0.31	0.46	10.695	381	< 0.001
Upper limb												
Shoulder pain	482	1.44	0.82	1.11	0.44	− 0.33	0.52	0.26	0.39	9.674	481	< 0.001
Elbow pain	94	1.31	0.69	1.10	0.39	− 0.21	0.39	0.10	0.33	3.648	93	< 0.001
Wrist pain	62	1.44	0.74	1.10	0.39	− 0.34	0.60	0.19	0.48	4.671	61	< 0.001
Hand pain	47	1.49	0.93	1.28	0.71	− 0.21	0.26	0.02	0.41	2.219	46	0.031
Non-responders (EQ-5D index change from baseline < 0.1)												
Lower limb												
Foot pain	32	2.00	0.88	1.81	0.74	− 0.19	0.23	-0.04	0.42	1.646	31	0.110
Ankle pain	49	1.98	0.90	1.59	0.81	− 0.39	0.45	0.13	0.64	3.065	48	0.004
Knee pain	253	1.92	0.88	1.60	0.78	− 0.32	0.39	0.23	0.41	7.061	252	< 0.001
Hip pain	113	1.85	0.86	1.64	0.74	− 0.21	0.27	0.08	0.34	3.285	112	0.001
Trunk and head												
Lumbar pain	301	1.63	0.80	1.50	0.77	− 0.13	0.17	0.05	0.20	3.416	300	0.001
Thoracic pain	34	1.12	0.41	1.21	0.54	0.09	− 0.19	− 0.25	0.07	− 1.139	33	0.263
Neck pain	181	1.27	0.56	1.31	0.61	0.04	− 0.08	− 0.11	0.03	− 1.236	180	0.218
Upper limb												
Shoulder pain	224	1.19	0.56	1.25	0.60	0.05	− 0.09	− 0.12	0.01	− 1.554	223	0.122
Elbow pain	56	1.07	0.32	1.09	0.35	0.02	− 0.05	− 0.10	0.06	− 0.444	55	0.659
Wrist pain	26	1.15	0.46	1.31	0.68	0.15	− 0.27	− 0.34	0.03	− 1.690	25	0.103
Hand pain	37	1.14	0.42	1.16	0.50	0.03	− 0.06	− 0.19	0.14	− 0.329	36	0.744

The self-care dimension showed significant improvement in EQ-5D responders across all symptom locations. Large improvements in the self-care dimension were seen for knee, hip, lumbar, neck, shoulder, elbow and wrist symptoms in EQ-5D responders, moderate improvement effects were seen for ankle, thoracic and hand symptoms and small improvements for foot symptoms (Table 3). EQ-5D non-responders showed trivial or small worsening effects for foot, ankle, knee, lumbar, thoracic, elbow and wrist symptoms. Significant increases (worsening) in the self-care dimension in non-responders were seen with hip and neck symptoms, although these changes showed small and trivial effect magnitudes, respectively. Pre-treatment self-care dimension scores ranged between 1.32 and 1.82 for EQ-5D responders and between 1.04 and 1.41 for EQ-5D non-responders.

**Table 3:** Pre- and post-treatment scores for the self-care dimension of the EQ-5D for patients that reported overall improvements in EQ-5D index and those that did not

	*n*	Pre-treatment		Post-treatment		Mean difference	*d*	95% confidence interval		*t*	df	*p*
		Mean	SD	Mean	SD			Lower	Upper			
Responders (EQ-5D index change from baseline > 0.1)												
Lower limb												
Foot pain	75	1.36	0.69	1.09	0.41	− 0.27	0.49	0.09	0.44	3.041	74	0.003
Ankle pain	141	1.32	0.69	1.02	0.19	− 0.30	0.68	0.19	0.41	5.426	140	0.000
Knee pain	538	1.51	0.78	1.07	0.30	− 0.44	0.81	0.38	0.50	13.834	537	0.000
Hip pain	188	1.57	0.72	1.10	0.34	− 0.47	0.89	0.37	0.56	9.886	187	0.000
Trunk and head												
Lumbar pain	690	1.75	0.85	1.12	0.37	− 0.63	1.03	0.58	0.70	20.337	689	0.000
Thoracic pain	94	1.32	0.51	1.05	0.23	− 0.27	0.73	0.15	0.38	4.670	93	0.000
Neck pain	382	1.56	0.75	1.10	0.37	− 0.46	0.82	0.39	0.53	13.215	381	0.000
Upper limb												
Shoulder pain	482	1.82	0.88	1.14	0.40	− 0.68	1.06	0.60	0.75	18.392	481	0.000
Elbow pain	94	1.51	0.77	1.05	0.31	− 0.46	0.85	0.31	0.61	6.090	93	0.000
Wrist pain	62	1.74	0.72	1.11	0.37	− 0.63	1.16	0.46	0.80	7.519	61	0.000
Hand pain	47	1.70	0.83	1.23	0.56	− 0.47	0.68	0.26	0.68	4.471	46	0.000
Non-responders (EQ-5D index change from baseline < 0.1)												
Lower limb												
Foot pain	32	1.28	0.68	1.41	0.71	0.13	− 0.19	− 0.30	0.05	− 1.438	31	0.161
Ankle pain	49	1.04	0.29	1.12	0.39	0.08	− 0.24	− 0.20	0.03	− 1.429	48	0.159
Knee pain	253	1.25	0.54	1.30	0.59	0.05	− 0.09	− 0.11	0.01	− 1.670	252	0.096
Hip pain	113	1.24	0.50	1.37	0.60	0.13	− 0.24	− 0.22	− 0.04	− 2.986	112	0.003
Trunk and head												
Lumbar pain	301	1.31	0.60	1.38	0.68	0.07	− 0.11	− 0.13	− 0.01	− 2.267	300	0.024
Thoracic pain	34	1.18	0.39	1.21	0.48	0.03	− 0.07	− 0.19	0.13	− 0.373	33	0.711
Neck pain	181	1.16	0.47	1.25	0.60	0.09	− 0.17	− 0.16	− 0.02	− 2.445	180	0.015
Upper limb												
Shoulder pain	224	1.41	0.70	1.38	0.62	− 0.03	0.05	− 0.04	0.10	0.749	223	0.454
Elbow pain	56	1.21	0.46	1.34	0.58	0.13	− 0.25	− 0.23	− 0.02	− 2.434	55	0.018
Wrist pain	26	1.23	0.51	1.27	0.53	0.04	− 0.08	− 0.28	0.20	− 0.328	25	0.746
Hand pain	37	1.19	0.46	1.16	0.37	− 0.03	0.07	− 0.16	0.21	0.298	36	0.768

The usual activities dimension showed large, significant, improvement across all symptom locations for EQ-5D responders (Table 4). In EQ-5D non-responders, significant improvements in the usual activities dimension were seen in patients with ankle, knee, hip, lumbar, neck, shoulder and elbow symptoms. These improvements were of moderate effect magnitude for elbow symptoms, and all other symptom locations showed small changes, except those with hand and wrist symptoms. Pre-treatment usual activities dimension scores ranged between 1.83 and 2.55 for EQ-5D responders and between 1.76 and 2.09 for EQ-5D non-responders.

**Table 4:** Pre- and post-treatment scores for the usual activities dimension of the EQ-5D for patients that reported overall improvements in EQ-5D index and those that did not

	*n*	Pre-treatment		Post-treatment		Mean difference	*d*	95% confidence interval		*t*	df	*p*
		Mean	SD	Mean	SD			Lower	Upper			
Responders (EQ-5D index change from baseline > 0.1)												
Lower limb												
Foot pain	75	2.55	1.03	1.21	0.50	− 1.34	1.75	1.11	1.55	11.987	74	0.000
Ankle pain	141	2.28	0.96	1.25	0.50	− 1.03	1.41	0.89	1.18	13.696	140	0.000
Knee pain	538	2.39	1.00	1.28	0.52	− 1.11	1.46	1.03	1.19	28.061	537	0.000
Hip pain	188	2.28	0.92	1.27	0.52	− 1.01	1.40	0.89	1.13	16.250	187	0.000
Trunk and head												
Lumbar pain	690	2.44	1.00	1.35	0.59	− 1.09	1.37	1.02	1.16	30.585	689	0.000
Thoracic pain	94	1.83	0.78	1.17	0.43	− 0.66	1.09	0.50	0.82	8.307	93	0.000
Neck pain	382	2.21	0.97	1.26	0.53	− 0.95	1.27	0.85	1.04	20.034	381	0.000
Upper limb												
Shoulder pain	482	2.33	0.96	1.32	0.59	− 1.01	1.30	0.94	1.10	25.315	481	0.000
Elbow pain	94	2.32	0.96	1.28	0.65	− 1.04	1.29	0.87	1.21	12.201	93	0.000
Wrist pain	62	2.26	0.83	1.31	0.56	− 0.95	1.37	0.75	1.15	9.389	61	0.000
Hand pain	47	2.47	0.97	1.60	0.77	− 0.87	1.00	0.60	1.14	6.476	46	0.000
Non-responders (EQ-5D index change from baseline < 0.1)												
Lower limb												
Foot pain	32	1.78	0.83	1.63	0.75	− 0.15	0.19	− 0.09	0.40	1.305	31	0.201
Ankle pain	49	1.94	0.80	1.65	0.78	− 0.29	0.37	0.09	0.48	2.954	48	0.005
Knee pain	253	2.09	0.80	1.78	0.78	− 0.31	0.39	0.22	0.40	6.580	252	0.000
Hip pain	113	1.92	0.80	1.73	0.79	− 0.19	0.24	0.05	0.32	2.767	112	0.007
Trunk and head												
Lumbar pain	301	1.96	0.84	1.77	0.89	− 0.19	0.22	0.10	0.28	4.170	300	0.000
Thoracic pain	34	1.76	0.70	1.56	0.79	− 0.20	0.27	− 0.05	0.46	1.646	33	0.109
Neck pain	181	1.86	0.79	1.59	0.81	− 0.27	0.34	0.16	0.39	4.665	180	0.000
Upper limb												
Shoulder pain	224	1.92	0.76	1.68	0.76	− 0.24	0.32	0.14	0.35	4.488	223	0.000
Elbow pain	56	2.05	0.70	1.61	0.76	− 0.44	0.60	0.26	0.64	4.696	55	0.000
Wrist pain	26	1.88	0.86	1.77	0.71	− 0.11	0.14	− 0.21	0.45	0.721	25	0.478
Hand pain	37	1.78	0.67	1.51	0.65	− 0.27	0.41	0.00	0.54	2.044	36	0.048

The pain/discomfort dimension showed a very large, significant, improvement across all symptom locations for EQ-5D responders (Table 5). EQ-5D non-responders showed significant reductions in the pain dimension for all sites of pain except thoracic, wrist and hand symptoms. Improvements of moderate effect magnitude in non-responders were observed for foot, ankle and elbow pain but all other symptom locations showed small or trivial improvements effects. Pre-treatment pain/discomfort dimension scores ranged between 2.54 and 2.97 for EQ-5D responders and between 1.89 and 2.44 for EQ-5D non-responders.

**Table 5:** Pre- and post-treatment scores for the pain/discomfort dimension of the EQ-5D for patients that reported overall improvements in EQ-5D index and those that did not

	*n*	Pre-treatment		Post-treatment		Mean difference	*d*	95% confidence interval		t	df	p
		Mean	SD	Mean	SD			Lower	Upper			
Responders (EQ-5D index change from baseline > 0.1)												
Lower limb												
Foot pain	75	2.97	0.93	1.49	0.64	− 1.48	1.88	1.26	1.70	13.705	74	0.000
Ankle pain	141	2.62	0.79	1.29	0.51	− 1.33	2.03	1.20	1.46	20.212	140	0.000
Knee pain	538	2.64	0.79	1.35	0.54	− 1.29	1.93	1.23	1.35	40.522	537	0.000
Hip pain	188	2.71	0.85	1.37	0.56	− 1.34	1.90	1.22	1.46	22.371	187	0.000
Trunk and head												
Lumbar pain	690	2.96	0.91	1.47	0.61	− 1.49	1.97	1.43	1.56	46.475	689	0.000
Thoracic pain	94	2.54	0.73	1.27	0.49	− 1.28	2.10	1.14	1.42	18.243	93	0.000
Neck pain	382	2.87	0.89	1.39	0.56	− 1.48	2.06	1.40	1.57	35.158	381	0.000
Upper limb												
Shoulder pain	482	2.77	0.85	1.37	0.58	− 1.40	1.96	1.33	1.47	39.615	481	0.000
Elbow pain	94	2.76	0.81	1.35	0.62	− 1.40	1.96	1.27	1.54	21.122	93	0.000
Wrist pain	62	2.74	0.75	1.40	0.56	− 1.34	2.06	1.19	1.48	18.461	61	0.000
Hand pain	47	2.83	1.07	1.51	0.66	− 1.32	1.53	1.07	1.57	10.486	46	0.000
Non-responders (EQ-5D index change from baseline < 0.1)												
Lower limb												
Foot pain	32	2.44	0.84	2.00	0.62	− 0.44	0.60	0.21	0.66	3.999	31	0.000
Ankle pain	49	2.35	0.69	2.00	0.61	− 0.35	0.53	0.16	0.54	3.663	48	0.001
Knee pain	253	2.36	0.82	2.11	0.73	− 0.25	0.32	0.17	0.33	5.855	252	0.000
Hip pain	113	2.43	0.74	2.19	0.71	− 0.25	0.34	0.12	0.38	3.827	112	0.000
Trunk and head												
Lumbar pain	301	2.45	0.76	2.23	0.78	− 0.21	0.28	0.13	0.29	5.109	300	0.000
Thoracic pain	34	2.24	0.78	2.06	0.69	− 0.18	0.24	− 0.01	0.36	1.977	33	0.056
Neck pain	181	2.44	0.76	2.17	0.74	− 0.27	0.35	0.16	0.37	5.066	180	0.000
Upper limb												
Shoulder pain	224	2.27	0.78	2.04	0.71	− 0.23	0.31	0.14	0.32	4.865	223	0.000
Elbow pain	56	2.41	0.73	2.04	0.57	− 0.38	0.58	0.23	0.52	5.029	55	0.000
Wrist pain	26	2.35	0.85	2.08	0.63	− 0.27	0.37	− 0.02	0.56	1.895	25	0.070
Hand pain	37	1.89	0.84	1.76	0.68	− 0.14	0.18	− 0.08	0.35	1.303	36	0.201

The anxiety/depression dimension showed a large, significant, improvement for foot, ankle, knee, hip, lumbar, thoracic, neck, elbow, wrist and hand symptoms in EQ-5D responders, with a moderate, significant improvement observed for shoulder symptoms (Table 6). EQ-5D non-responders showed no change in the anxiety/depression dimension, with only trivial effect magnitudes being observed for all symptom locations. Pre-treatment anxiety/depression dimension scores ranged between 1.37 and 1.78 for EQ-5D responders and between 1.11 and 1.41 for EQ-5D non-responders.

**Table 6:** Pre- and post-treatment scores for the anxiety/depression dimension of the EQ-5D for patients that reported overall improvements in EQ-5D index and those that did not

	*n*	Pre-treatment		Post-treatment		Mean difference	*d*	95% confidence interval		*t*	df	*p*
		Mean	SD	Mean	SD			Lower	Upper			
Responders (EQ-5D index change from baseline > 0.1)												
Lower limb												
Foot pain	75	1.73	0.92	1.08	0.32	− 0.65	1.05	0.45	0.86	6.448	74	0.000
Ankle pain	141	1.53	0.77	1.04	0.19	− 0.50	1.04	0.37	0.62	7.835	140	0.000
Knee pain	538	1.57	0.81	1.06	0.28	− 0.52	0.95	0.45	0.59	15.066	537	0.000
Hip pain	188	1.57	0.83	1.06	0.26	− 0.51	0.94	0.40	0.62	9.435	187	0.000
Trunk and head												
Lumbar pain	690	1.78	0.97	1.11	0.40	− 0.67	0.98	0.60	0.74	19.387	689	0.000
Thoracic pain	94	1.48	0.79	1.05	0.27	− 0.43	0.81	0.28	0.57	5.932	93	0.000
Neck pain	382	1.66	0.87	1.10	0.39	− 0.55	0.88	0.47	0.64	13.489	381	0.000
Upper limb												
Shoulder pain	482	1.60	0.93	1.10	0.39	− 0.50	0.76	0.42	0.58	12.492	481	0.000
Elbow pain	94	1.47	0.80	1.03	0.18	− 0.44	0.89	0.28	0.60	5.396	93	0.000
Wrist pain	62	1.37	0.61	1.03	0.18	− 0.34	0.86	0.19	0.49	4.452	61	0.000
Hand pain	47	1.72	0.97	1.15	0.47	− 0.57	0.80	0.34	0.81	4.919	46	0.000
Non-responders (EQ-5D index change from baseline < 0.1)												
Lower limb												
Foot pain	32	1.25	0.57	1.25	0.72	0.00	0.00					
Ankle pain	49	1.24	0.56	1.31	0.85	0.06	− 0.09	− 0.24	0.12	− 0.685	48	0.497
Knee pain	253	1.35	0.67	1.31	0.70	− 0.04	0.06	− 0.03	0.11	1.209	252	0.228
Hip pain	113	1.24	0.52	1.28	0.57	0.04	− 0.08	− 0.13	0.04	− 1.000	112	0.319
Trunk and head												
Lumbar pain	301	1.40	0.73	1.45	0.84	0.05	− 0.07	− 0.12	0.01	− 1.558	300	0.120
Thoracic pain	34	1.21	0.54	1.29	0.58	0.09	− 0.16	− 0.27	0.09	− 1.000	33	0.325
Neck pain	181	1.41	0.74	1.38	0.81	− 0.03	0.04	− 0.04	0.11	0.884	180	0.378
Upper limb												
Shoulder pain	224	1.25	0.62	1.22	0.65	− 0.02	0.04	− 0.05	0.09	0.610	223	0.542
Elbow pain	56	1.36	0.67	1.43	0.71	0.07	− 0.10	− 0.24	0.10	− 0.851	55	0.399
Wrist pain	26	1.27	0.45	1.31	0.62	0.04	− 0.07	− 0.11	0.11	0.000	36	1.000
Hand pain	37	1.11	0.31	1.11	0.39	0.00	0.00	− 0.22	0.22	0.000	31	1.000

## Discussion

The aim of this study was to determine how community-based physiotherapy treatment for musculoskeletal injury was related to change in the five dimension scores of the EQ-5D HRQoL outcome measure. A main finding was that the pain/discomfort dimension of the EQ-5D outcome reduced across all symptom locations, but this reduction did not equate to an improvement in EQ-5D index for all patients. Therefore, during a course of physiotherapy, pain reduction often occurs but EQ-5D will not improve in total score. The anxiety/depression dimension mean score improved in patients that showed an increase in EQ-5D index of greater than 0.1, suggesting that anxiety and depression could be a key dimension and status linked to the potential to improve EQ-5D and hence HRQoL.

EQ-5D responders had higher pre-treatment pain/discomfort (2.54–2.97) than EQ-5D non-responders (1.89–2.44), but showed reduced post-treatment pain/discomfort (1.27–1.51) when compared to EQ-5D non-responders (1.76–2.23). Of all five dimensions, pain/discomfort has been reported as being the most frequently problematic dimension across a range of musculoskeletal conditions [18]. The fact that community-based physiotherapy was associated with at least small reductions in the pain/discomfort dimension in all patients, regardless of whether they reported overall improvements in HRQoL is encouraging. However, this would need to be confirmed via a randomised controlled trial using validated joint-specific pain outcomes.

The anxiety/depression dimension reduced in EQ-5D responders, with large effect magnitudes in symptom locations except the shoulder (moderate effect). EQ-5D non-responders reported no change in anxiety/depression, with trivial effect magnitudes across all symptom locations. Pre-treatment anxiety/depression dimension scores were higher in EQ-5D responders than EQ-5D non-responders. Following treatment, the anxiety/depression dimension was lowest in EQ-5D responders. These data suggest that if patients present with higher levels of anxiety/depression, they are, in fact, more likely to show improved HRQoL than those who present with less anxiety/depression. This finding contradicts the findings of Bergbom et al [19], who observed a lack of response to treatment in patients who presented with depressed mood. Previous studies have also shown high levels of depression to be linked to poor adherence to exercise rehabilitation programmes [20–23]. Before treatment, the mean anxiety/depression score in EQ-5D non-responders ranged between 1.11 and 1.40, compared to between 1.37 and 1.78 in EQ-5D responders.

The anxiety/depression dimension scores at presentation for EQ-5D responders are still relatively low, so may not represent true clinical depression in most patients in a physiotherapy cohort compared to those reported in previous studies as showing poor outcome and poor adherence to treatment. The lower levels of depression in the EQ-5D non-responders could be considered as very low, though Shaw et al. [20] found that intervention adherence was poor in osteoarthritic patients with very low depression as well as with very high depression contrary to our findings. Further research is warranted to look at the relationship between treatment adherence and HRQoL scores at presentation.

The mobility dimension showed the greatest improvements in EQ-5D responders that received treatment for lower limb and low back conditions, showing large effect magnitudes. In EQ-5D responders receiving treatment at other symptom locations, only small to moderate improvements in the mobility dimension were observed. In EQ-5D non-responders, improvements with small effect magnitudes were observed for lower limb and wrist symptoms, and only trivial effects for trunk and upper limb symptom locations. Mobility is sometimes assessed using functional tests such as the 6-min walk test [24], which naturally make more use of the legs and trunk (load bearing) than the upper limbs (non-load bearing). The questions asked in the EQ-5D outcome specific to the mobility dimension are also specific to walking [4]. It is not surprising, therefore, that changes to the mobility dimension of the EQ-5D outcome were most associated with lower limb and lumbar pain. These findings suggest that evaluation of mobility status should only be considered in patients presenting with lower limb or low back symptoms.

The self-care dimension improved in EQ-5D responders, with moderate to large effect magnitudes observed in all symptom locations except the foot (small effect magnitude). In EQ-5D non-responders, most symptom locations showed no change in the self-care dimension, and patients receiving treatment for ankle, hip and elbow symptoms reported small declines in the self-care dimension. When completing the EQ-5D outcome, patients indicate their ability to wash themselves and to get dressed [4]. Having full range of motion at the hip and elbow will be key to washing and bending to be able to reach the feet, and ankle pain will likely impact on a patient’s ability to put on their socks and shoes. These data suggest that, for patients with ankle, hip and elbow symptoms, self-care is an important factor in whether a patient’s overall HRQoL improves.

In EQ-5D responders, improvements in the usual activities dimension (e.g. work, study, housework, family or leisure activities [4] with large magnitudes of effect reported across all symptom locations. In EQ-5D non-responders, usual activities were seen to improve with small effect magnitude for most symptom locations, with the exception of the elbow (moderate effect), foot (trivial effect) and wrist (trivial effect). This finding is similar to that discussed for the mobility dimension. Walking (the activity referred to in the mobility dimension question) could be considered one of the most prevalent “usual activities” that many patients will perform. In fact, Simpson et al [25] reported that walking was the most frequently reported physical activity amongst US adults in 2000, with 46% primarily walking for a minimum of 30 min, five times per week, as a leisure-time physical activity. This figure is likely to be slightly lower in the UK as Adams [26] reported only 34% of UK adults complete this duration/frequency of physical activity each week. As such, it could be expected that the usual activities dimension would make a similar contribution to overall HRQoL as the mobility dimension, as they are both functional dimensions within the EQ-5D outcome measure [27].

The data suggest that pain/discomfort and anxiety/depression show the greatest change in EQ-5D responders compared to EQ-5D non-responders. Interestingly, EQ-5D responders reported pre-treatment scores for the pain/discomfort dimension and the anxiety/depression dimension that were higher (worse) than in EQ-5D non-responders. These data suggest that those patients presenting with increased pain/discomfort and/or anxiety/depression may have the greatest capacity for improving their overall HRQoL. It must be noted, however, that the majority of patients reported only slight to moderate pain/discomfort and either no or slight anxiety/depression. This suggests that the EQ-5D dimensions may not be sufficiently sensitive to identify improvements in HRQoL in these patients. Herdman et al. [27] reported on the development of the EQ-5D five-level outcome (as used here) which attempted to reduce ceiling effects over the three-level version of the outcome. The current data suggest that even the five-level version of this outcome shows a ceiling effect.

The fact that many patients reported no pain/discomfort on the EQ-5D pain/discomfort dimension (i.e. score of 1), especially with the lack of improvement in overall HRQoL for many of those patients, raises the question as to whether it is economically appropriate for them to receive intensive physiotherapy. This would be in line with the approach of the STarT Back stratification tool for low back pain [28]. It could be that patients reporting no or low levels of pain/discomfort and anxiety/depression would see just as good outcomes in terms of HRQoL from receiving minimal intervention. Black [2], however, cautioned against HRQoL outcomes being used to ration care, suggesting that other factors, such as long-term outcomes of the specific disease, must also be considered. The assessment of HRQoL is suggested as being more useful in clinical decision making compared to disease-specific outcomes when it forms a part of a patient’s early evaluation [3]. However, quality of life is difficult to interpret as it is highly individual [3]. Quality of life should, therefore, be measured by the clinician in order that it is appropriately interpreted [2] and is used in a proportionate way to aid clinical goal setting [3].

It must be remembered that the individual EQ-5D dimensions are not validated as individual outcome measures. Multi-dimensional HRQoL measures are also not as effective as joint-specific outcomes in the evaluation of treatment outcome [3]. The present data point, however, to the importance of assessing pain/discomfort and anxiety/depression using a validated joint-specific outcome as part of a randomised controlled trial to assess the effectiveness of community-based physiotherapy for musculoskeletal conditions. Future studies should use validated outcomes for pain/discomfort and anxiety/depression in order to fully evaluate the effectiveness of community-based physiotherapy for musculoskeletal conditions. Whilst the EQ-5D dimensions suggest improvements over the time course of treatment, it is not possible from the current data to confidently say that this improvement in HRQoL was due to the physiotherapy treatment due to the retrospective nature of the data (i.e. no control group), and there is potential that changes in HRQoL reported could be due to the natural time course of healing. The data presented here should be used, therefore, to inform the design of a randomised controlled trial to fully evaluate the effectiveness of community-based physiotherapy. Such a trial should use specific pain/discomfort and anxiety/depression outcomes in this patient group to investigate the relevance of these dimensions to HRQoL and clinical outcomes.

## Conclusions

In conclusion, following a community-based physiotherapy intervention for musculoskeletal conditions, the pain/discomfort dimension scores improved across all patients and all symptom locations yet was not necessarily linked to improved HRQoL. Patients that showed the greatest improvement in overall EQ-5D index presented with worse pain/discomfort and/or anxiety/depression, as measured by the respective EQ-5D dimensions. Clinicians should not, therefore, assume that a patient with worse pain and anxiety/depression is unlikely to respond to treatment, as they may, in fact, show the best outcomes in terms of HRQoL. The EQ-5D index, however, is not sensitive enough for the evaluation of patient presenting with low levels of pain/discomfort and/or anxiety/depression, so is perhaps not a suitable tool for sole use in patient management and service evaluation. The findings suggest that additional validated pain and anxiety/depression outcomes could be used to compliment EQ-5D to better capture the experiences of patients and identify patients that might respond best to treatment.
